# Fusing Semantic and Structural Features for Code Error Detection

**DOI:** 10.3390/e27121229

**Published:** 2025-12-04

**Authors:** Yiwen Zhang, Wei Liu, Fazhong Jiang, Jiquan Ma, Jingtai Cao

**Affiliations:** 1 National Astronomical Observatories, Chinese Academy of Sciences, Beijing 100101, China; 2 Department of Communication Engineerings, Jilin University, Changchun 130012, China; 3 School of Computer and Big Data, Heilongjiang University, Harbin 150080, China; 4 Changchun Changguang Orion Optoelectronic Technology Co., Ltd., Changchun 130033, China; 5 Changchun Institute of Optics, Fine Mechanics and Physics, Chinese Academy of Sciences, Changchun 130033, China

**Keywords:** code error detection, large language models, graph neural networks

## Abstract

Large Language Models of the Transformer architecture display great promise in automated code error detection based on their strength in processing sequential data. Nevertheless, their efficacy could be further improved by addressing the inherent weakness in handling structural code dependencies. In response to this, we introduce a novel model that integrates the semantic comprehension power of RoBERTa with the structural learning strength of Graph Neural Networks. This model aims to detect the most common categories of programming faults in the form of runtime errors, index errors, and import/module errors. Experimental evaluation has demonstrated that the hybrid model, utilizing a proper fusion technique, outperforms other models in terms of accuracy and robustness. The introduced mechanism leads to numerical benefits, improving test accuracy by 1.75% over competitive baseline.

## 1. Introduction

Code error detection is a key component in software development, which helps quickly identify and fix errors, thereby improving software quality and efficiency. However, conventional approaches are unable to keep up with scale and diversity in large codebases. Static code analysis and dynamic debugging are subject to severe limitations. Static code analysis checks syntax and semantics without being able to catch runtime faults like memory leaks or concurrency faults. Dynamic debugging, on the other hand, can identify runtime faults more reliably but is a time-consuming and inefficient process and not feasible for large projects. Due to this, Machine Learning (ML) and Deep Learning (DL) approaches have been proposed as viable alternatives to improve fault detection capability. Especially Natural Language Processing (NLP) models like BERT [1] and GPT [2], as well as other pre-trained models, can understand the semantics of code just like understanding human language. These models can learn to recognize patterns, relationships, and structures in code through training on large-scale code repositories, thereby identifying both simple and complex errors, while promising, these models also suffer from limitations in handling long-range dependencies and dynamic behaviors of large-scale systems. Thus, we need novel methods that bring together multiple modalities to tackle the challenge of code fault detection in real-world software engineering in a better way.

In response to the limitations of traditional techniques, numerous studies have explored the use of deep learning in code analysis tasks. Early approaches borrowed architectures from NLP, such as Convolutional Neural Networks (CNNs) and Recurrent Neural Networks (RNNs), to learn semantic features and contextual patterns from source code [3,4]. Models like DeepBugs [5] and BugBuster [6] demonstrated that neural networks can detect common programming mistakes and even binary-level errors by analyzing large-scale code data.

More recently, the rise of large pre-trained Large Language Models (LLMs) such as BERT [1], RoBERTa [7], and CodeBERT [8] has significantly improved the performance of code understanding and error detection tasks. These models learn syntactic and semantic information through large-scale self-supervised training. To further enhance model effectiveness, recent studies have shown that the use of LLMs and Graph Neural Networks (GNNs) in conjunction is a highly effective approach in tasks such as text classification, leveraging the synergy of semantic and structural reasoning.

This effectiveness is successfully applied to code analysis, where semantic context and structural patterns also play a decisive role. For example, the WitheredLeaf framework [9] leverages GPT-4 [2] alongside static analysis tools, emphasizing the importance of extracting rich structural and semantic information from code for tasks like entity inconsistency detection.

Based on this foundation, this paper proposes a novel model that combines RoBERTa [7] and the Graph Isomorphism Network (GIN) for code error detection. With the combination of multiple levels of structural and semantic information, this model can efficiently identify global code dependencies as well as local context. The structural relationships from code snippets are modeled by GIN while token-level semantic representations are encoded by RoBERTa. The model with the fusion of these features gain the ability to detect a wide range of code issues, including semantic inconsistencies, logical errors, and potential security vulnerabilities.

To summarize, the main contributions of this work are as follows:

- We propose a novel framework that integrates LLMs and GNN for processing code and detecting multiple types of errors. This method fully utilizes the powerful semantic understanding ability of LLM and the structural reasoning ability of GNN, which can simultaneously capture the semantic context and structural patterns in the code, achieving accurate detection of diverse error types.
- We systematically studied different fusion strategies of GNN and LLMs, and deeply analyzed the impact of various fusion methods on the structural representation ability of the model. The research results indicate that designing an effective fusion mechanism plays a key role in leveraging the advantages of semantic and structural information and improving the effectiveness of code analysis.
- We introduced the PytraceBugs dataset for code error classification and verified through extensive experiments that our proposed model with cross-modal fusion outperforms other models, including model that relies solely on RoBERTa. The experimental results show that a reasonable fusion strategy can improve the understanding ability of code information.

Experimental results demonstrate that this model with appropriate fusion strategy not only outperforms traditional model but also provides a scalable and robust solution for error detection in complex, real-world code scenarios.

The remainder of the paper is organized as follows. Section 2 reviews related work on code error detection, LLMs, GNNs, and their integration. Section 3 introduces the details of RoBERTa with GNN model. Section 4 presents experimental settings, results, and analysis. Finally, Section 5 gives the conclusion of this paper.

## 2. Related Work

This section reviews prior work related to code error detection, focusing on approaches utilizing LLMs, GNNs, and hybrid methods that integrate both semantic and structural representations.

### 2.1. Code Error Detection

In the field of Natural Language Processing, machine learning, especially deep learning, has achieved remarkable success. Traditional text processing techniques, which include rule-based models and shallow machine learning methods, can manage simple tasks but struggle to handle complex semantics and long-distance dependencies. Machine learning has made significant advancements in tasks such as text classification, sentiment analysis, machine translation, and question answering. For example, CNNs have demonstrated outstanding performance in text classification by effectively capturing local features within sentences [10]. In sentiment analysis, RNNs have been applied to process syntactic structures and successfully identify sentiment [11]. In machine translation, the introduction of attention mechanisms has greatly improved the quality of neural machine translation models, particularly in aligning source and target languages [12]. In addition, deep learning has improved question-answering systems to learn large knowledge bases and effectively pose answers to open-domain questions [13].

Traditional methods, such as static analysis and dynamic debugging, face limitations. Static and dynamic methods of debugging work by statically analyzing the code and detecting syntax and semantic errors but fail in detecting runtime faults such as memory leaks or concurrency-related problems [14]. Dynamic debugging is inefficient and expensive to use, particularly in large-scale projects [15]. These traditional methods often fail to detect complex logical errors such as deadlocks or race conditions [16]. Deep learning methods, particularly neural network-based models, can automatically learn complex code patterns and semantic information from large-scale code data. For example, studies have shown that Deep Neural Networks (DNN [17]), CNNs [18,19], RNNs [20,21] can be used for code error detection, enhancing error recognition through large-scale code analysis [3,4]. In recent years, an increasing number of deep learning models have been applied to code error detection tasks. For instance, DeepBugs [5] identifies common errors in code, such as null pointer exceptions and array out-of-bounds errors, by analyzing patterns in code variable names using neural networks. Meanwhile, BugBuster [6] leverages neural networks to learn the deep semantic information of code, effectively detecting binary errors and improving automated error recognition capabilities.

### 2.2. LLMs in Code Error Detection

In recent years, LLMs have become widely popular for their ability to process and generate human-like text across various domains. Prominent examples of LLMs include GPT-4 [2], which is good at natural language generation and reasoning; Claude [22], which is developed by Anthropic with emphasis on safety and contextual understanding; PaLM [23], a Google powerful model geared towards various tasks in natural languages; and LLaMA [24], an efficient open-source model optimized for research and practical applications. As a result, LLMs have made significant progress in code understanding and error detection. Pre-trained models based on the Transformer architecture [25], such as BERT [1], RoBERTa [7], and CodeBERT [8], capture both syntax and semantic features of code through large-scale pretraining, enabling their effective application in code error detection, fault localization, and other tasks. CodeBERT [8] considers both the language features of code and natural language understanding during pretraining, demonstrating excellent performance in error detection tasks across multiple programming languages.

RoBERTa [7], as an improved version of BERT, enhances performance in code error detection by leveraging larger datasets and optimized pretraining strategies. Research by Alrashedy et al. [26] further demonstrates that LLMs outperform traditional methods in binary error classification tasks. Despite their strengths in capturing semantic information and contextual representation, LLMs remain challenged in processing complex reasoning and structure dependencies, as well as modeling intricate relationships such as function call dependencies and control flow dependencies.

### 2.3. GNNs in Code Error Detection

GNNs [27] model code based on graph structures [28] and learn structural dependencies such as function call relationships, data dependencies, and control flow, which play critical roles in the detection of complex errors. Compared to traditional sequence-based models (e.g., LSTMs [21] and Transformers [25]), GNNs effectively represent the structural information of code through nodes (code elements, such as variables and functions) and edges (representing relationships such as data dependencies and control flow). This enables GNNs to demonstrate significant strengths in handling complex code logic, particularly in vulnerability detection, error localization, and code optimization.

The Devign method proposed by Zhou et al. [29], which combines GCN [30] with Abstract Syntax Trees (AST [31,32]), effectively identifies vulnerabilities in programs. It is revealed from research that GNNs can learn automatically from code structure and enhance error detection accuracy with the help of the mechanism of propagation in graphs. Other related studies, such as Briem et al. [33], proposed using Program Dependency Graphs (PDGs [34]) for advanced bug detection, while Lam et al. [35] combined Control Flow Graphs (CFGs [36]) to improve bug localization in programs. These research works indicate that the use of graph neural networks enables models to cope with the code’s complex structure and logic with ease, thereby enhancing the efficiency and accuracy of error detection.

### 2.4. Integration of LLMs and GNNs

While each LLM and GNN possesses its particular strengths in code error detection, each also possesses its own weaknesses. LLMs are strong at representing semantic information while being poor at encoding complex structural relationships, while GNNs are capable of representing structural information well but fall short in representing code’s contextual and semantic nature. Consequently, recent studies have been examining the combination of LLMs and GNNs to leverage the strengths of both models, thereby enhancing accuracy and comprehensiveness in error detection.

As an example, the use of the combination of LLMs and GNNs has shown remarkable performance in text processing tasks with the successful application of semantic comprehension and structural reasoning together. At the same time, works such as the WitheredLeaf framework proposed by Chen et al. [9] emphasize the importance of extracting structured information during code analysis. WitheredLeaf leverages GPT-4 alongside static analysis tools and lightweight code completion models to detect entity inconsistency bugs, highlighting how modular design and structural insights enhance error detection. These papers illustrate that the integration of LLMs and GNNs for code error classification is promising since the interaction of semantic and structural information helps to overcome limitations in the use of single model and provides more comprehensive solutions in codebase error evaluation and detection.

## 3. Methodology

### 3.1. Network Overview

**Figure 1:**
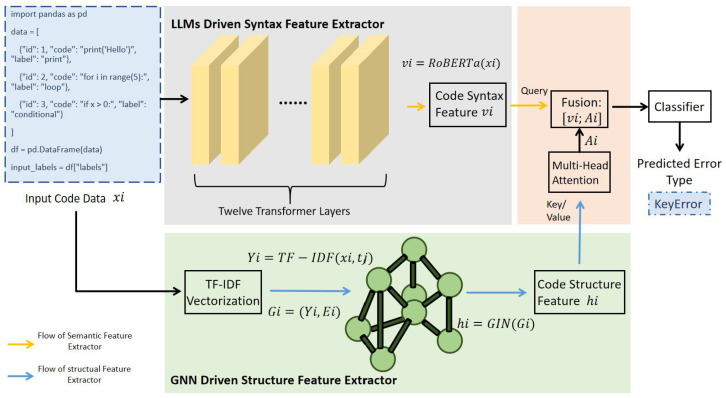
The Architecture of the model. The LLMs driven Syntax feature extractor component is shown in the gray part, while the GNN driven Structure feature extractor component is shown in the light green part. This picture shows the model with the cross-modal fusion which is shown in the light orange part.

To effectively classify code errors, the study proposes a model that combines the semantic information extracted by the RoBERTa model with the structural information extracted by a GNN. This deployment of two features allows the model to learn more about the structure and content of the code. The architecture of the model is shown in the picture below (Figure 1).

#### 3.1.1. Semantic Feature Extraction with RoBERTa

RoBERTa, a pre-trained language model based on the Transformer architecture, is employed to extract semantic features from code snippets. For a given code snippet $x_{i}$, it is first tokenized using a Byte Pair Encoding (BPE) tokenizer provided by RoBERTa to form an input token sequence. This sequence is then processed by the RoBERTa model to produce a semantic embedding $v_{i}\in R^{d}$, where *d* is the dimension of the embedding:

$$v_{i}=\mathrm{RoBERTa}\left(x_{i}\right).$$

This semantic vector $v_{i}$ can capture contextual relationships in code snippets, enabling the model to identify errors based on subtle differences in syntax and semantics. The semantic representation of the entire code snippet is obtained by aggregating the model’s understanding of each tag, thereby forming an abstract expression of the overall meaning of the code snippet.

#### 3.1.2. Structural Feature Extraction with GNN

To address the limitations of semantic models in capturing code structure, this study employs a Graph Neural Network, specifically the Graph Isomorphic Network.

Code snippets are transformed into graph representations $G_{i}=\left(Y_{i},E_{i}\right)$, where $Y_{i}$ denotes nodes (e.g., functions, variables, modules) $E_{i}$ denotes edges (e.g., dependencies, call relationships)

In this study, the dataset does not provide explicit structures.Therefore, to derive a graph representation suitable for GNN processing, we construct a lightweight context-based graph for each batch of code snippets.Specifically, given a batch $\left(x_{1},x_{2},\ldots ,x_{m}\right)$, each snippet is treated as a node, and edges are created between nodes that appear within a fixed contextual window:

E={(k,j)∣|k−j|≤5,j≠k}.

This sliding-window strategy captures local structural and stylistic proximity among code snippets, reflecting the intuition that adjacent fragments often share related patterns. To ensure that each node preserves its original information during message passing, self-loop edges (k,k) are added for all nodes.

The GIN aggregates features from each node and its neighbors through multiple layers of graph convolutions, generating a structural embedding $h_{i}\in R^{d}$:

$$h_{i}=\mathrm{GIN}\left(G_{i}\right).$$

To enhance the accuracy of GNN-based structural information extraction, this study preprocesses the textual content of the code snippets using the Term Frequency–Inverse Document Frequency (TF-IDF) [37] method. TF-IDF measures the importance of a specific feature (e.g., word, symbol) within a code snippet. For a code snippet $x_{i}$, the TF-IDF value for the *j*-th feature is defined as:

$$\mathrm{TF}\text{-}\mathrm{IDF}\left(x_{i},t_{j}\right)=\mathrm{TF}\left(t_{j},x_{i}\right)\cdot \log\frac{N}{\mathrm{DF}(t_{j})},$$

where $\mathrm{TF}(t_{j},x_{i})$ represents the term frequency of $t_{j}$ in code snippet $x_{i}$, $\mathrm{DF}(t_{j})$ is the number of code snippets containing $t_{j}$, and *N* is the total number of code snippets.

The initial feature vector ${\tilde{v}}_{i}$ generated by TF-IDF is used as the attributes for the nodes $V_{i}$ in the GNN model. The GIN further aggregates these node features through neighbor information, updating each node’s representation:

$$h_{i}^{\left(l+1\right)}=\mathrm{MLP}\left(\left(1+\epsilon \right)\cdot h_{i}^{\left(l\right)}+\sum_{j\in N(i)}h_{j}^{\left(l\right)}\right),$$

where $h_{i}^{\left(l\right)}$ represents the features of node *i* at layer *l*, N(i) denotes the set of neighboring nodes of *i*, ϵ is a learnable weight parameter, and MLP is a Multi-Layer Perceptron for feature mapping.

Finally, the structural embedding $h_{i}$ generated by GIN encodes the dependency relationships, call structures, and other structural information in the code, providing critical support for subsequent classification tasks.

#### 3.1.3. Feature Fusion and Classification

In order to fully utilize the semantic and structural information of the code, this study proposes a cross-modal fusion model that synergistically models RoBERTa and GIN based on Transformer. RoBERTa extracts contextual semantic representations of source code, while GIN models graph structural features such as dependencies and call structures in the code.

In this study, cross-modal attention fusion achieves effective interaction between the semantic vectors output by RoBERTa and the structural vectors extracted by GIN by introducing Multi Head Attention (MHA) [25] mechanism.

The cross-modal attention is defined as:

$$\mathrm{Attention}\left(Q,K,V\right)=\mathrm{Softmax}\left(\frac{QK^{⊤}}{\sqrt{d}}\right)V,$$

where $Q=v_{i}$, $K={\tilde{h}}_{i}$, $V={\tilde{h}}_{i}$. Applying multi-head attention gives:

$${\hat{h}}_{i}=\mathrm{MultiHead}\left(v_{i},{\tilde{h}}_{i},{\tilde{h}}_{i}\right),$$

where $x_{i}$ is the input code snippet, $v_{i}=\mathrm{RoBERTa}\left(x_{i}\right)\in R^{d}$ is the semantic vector extracted by RoBERTa, $h_{i}=\mathrm{GIN}\left(x_{i}\right)\in R^{d^{\prime }}$ is the structural vector extracted by GIN (after TF-IDF initialization), ${\tilde{h}}_{i}=W_{h}h_{i}\in R^{d}$ is the structural vector projected to the same dimensional space as $v_{i}$ through a linear layer.

In terms of specific implementation, firstly, the structural vector $h_{i}$ is transformed into the same dimension as the RoBERTa output vector through linear mapping, obtaining ${\tilde{h}}_{i}$. Subsequently, we use the semantic vector $v_{i}$ extracted by RoBERTa as the Query, and the mapped structural vector ${\tilde{h}}_{i}$ simultaneously input as Key and Value vectors into the multi-head attention module. This attention mechanism dynamically assigns weights based on the similarity between the Query and Key, and weights and sums the Value vectors to obtain the attention results of semantic modality on structural modality. Ultimately, the structure of attention output represents ${\hat{h}}_{i}$, regarded as the result of semantic information filtering and reconstructing structural information, it can efficiently extract the most relevant structural features to the current semantic context. This mechanism not only breaks down the information barriers between modalities, but also dynamically models the correlation between semantics and structure, achieving precise and controllable information fusion.

The final joint representation is obtained by concatenating $v_{i}$ and ${\hat{h}}_{i}$:

$$z_{i}=\left[v_{i};{\hat{h}}_{i}\right]\in R^{2d},$$

where ${\hat{h}}_{i}$ is the attention-enhanced structural representation aligned to the semantic context.

The concatenated vector is then passed through a fully connected layer followed by a softmax activation function to classify the error type:

$${\hat{y}}_{i}=\mathrm{Softmax}\left(Wz_{i}+b\right),$$

where *W* and *b* are trainable parameters of the classifier.

By integrating semantic and structural information, the model achieves a more comprehensive understanding of the code, enabling it to detect errors with higher accuracy and robustness.

### 3.2. Loss Function and Class Imbalance Handling

To mitigate the effect of class imbalance observed in the dataset—particularly the overrepresentation of index errors—we applied a weighted cross-entropy loss during model training. The weight assigned to each class $c_{j}$ was computed as:

(1) $$w_{j}=\frac{N}{k\cdot N_{j}},\;j=1,2,\ldots ,k,$$

where *N* is the total number of training samples, $N_{j}$ is the number of samples in class $c_{j}$, and *k* is the total number of classes. These weights were incorporated into the cross-entropy loss function as follows:

(2) $$L=-\frac{1}{N}\sum_{i=1}^{N}w_{y_{i}}\cdot \log P\left(y_{i}|x_{i}\right),$$

where $P(y_{i}|x_{i})$ denotes the predicted probability for the true label $y_{i}$. This weighting strategy follows the approach of [38] and encourages the model to allocate greater importance to underrepresented classes during optimization.

## 4. Experiment

### 4.1. Experimental Setup

#### 4.1.1. Dataset

This study utilizes the PyTraceBugs [39] dataset, which comprises real-world Python 3 code snippets and their corresponding error type labels. Each sample in the dataset is defined as:

$$D={\left\{\left(x_{i},y_{i}\right)\right\}}_{i=1}^{N},$$

where: $x_{i}$ represents the code snippet, and $y_{i}\in \left\{c_{1},c_{2},\ldots ,c_{k}\right\}$ denotes the associated error type label. Here, k=3, which includes runtime errors, index errors, and import/module errors.

To evaluate the model’s performance, the dataset was divided into a training set ($D_{\mathrm{train}}$), a validation set ($D_{\mathrm{val}}$), and a test set ($D_{\mathrm{test}}$), satisfying the conditions:

$$D_{\mathrm{train}}\cup D_{\mathrm{val}}\cup D_{\mathrm{test}}=D,\;D_{\mathrm{train}}\cap D_{\mathrm{val}}=\emptyset ,\;D_{\mathrm{val}}\cap D_{\mathrm{test}}=\emptyset .$$

The dataset was allocated in proportions of 70% for the training set, 15% for the validation set, and 15% for the test set:

$$p_{\mathrm{train}}=0.7,\;p_{\mathrm{val}}=0.15,\;p_{\mathrm{test}}=0.15.$$

This partitioning ensures that the model can be optimized on the validation set and its generalization ability can be assessed on the test set.

#### 4.1.2. Implemetation Details

In terms of model architecture, this work employs a pre-trained Transformer-based RoBERTa model to retrieve semantic information from code snippets. This enables the model to comprehend the semantic features present in the code and perform an initial classification task. Based on this model, GIN is introduced to capture structural information, including dependency relationships and control flows within code. The GIN forms a graph representation of code and embeds these structural attributes into high-dimensional vectors. Finally, the semantic vectors extracted by RoBERTa are fused with the structural vectors produced by GIN through feature concatenation and are passed into a fully connected classification layer to complete the prediction task. To further enhance the transmission of structural information and address potential issues, a cross-modal fusion technique is integrated to the combination of RoBERTa with GIN model, improving overall training stability and model performance.

Several key hyperparameters were tuned, including the learning rate (1 × 10^−5^, 2 × 10^−5^, 3 × 10^−5^, 5 × 10^−5^), batch size (8, 16, 32), and weight decay (0.01, 0.05, 0.1). Each configuration was trained for a short validation cycle, and the best-performing values on the validation set were selected for the final model. Each configuration was trained for a short validation cycle, and the best-performing values on the validation set were selected for the final model. The training and optimisation process is carried out with the use of the AdamW [40] optimiser with a starting learning rate of $3\times 10^{-5}$ and a weight decay of 0.05 to avoid overfitting. The batch size is set to 16 to maintain consistency and stability during both training and evaluation phases. Training is split into two stages: a warm-up phase of 10,000 steps, during which the learning rate is smoothly scaled to achieve stable convergence, followed by a regular training phase with a maximum of 100,000 steps. To ensure the controllability and stability of the training process, logs are recorded every 500 steps, while model evaluations and checkpoints are saved every 10,000 steps. Additionally, mixed precision training is employed to enhance computational efficiency and reduce memory consumption.

### 4.2. Evaluation Matrices

Model performance is measured based on three main metrics: accuracy, macro-averaged metrics, and loss function analysis. Accuracy assesses the general correctness of the classification process, whereas macro-averaged metrics measure the model’s capacity to classify under class imbalance and achieve fair detection of all the error types, particularly minority classes. Loss function analysis is employed to monitor optimisation progress and convergence trends during training. In addition to the core evaluations, this study further analyzes the overall performance of the model with the impact of different fusion strategies.

### 4.3. Experimental Results

**Figure 2:**
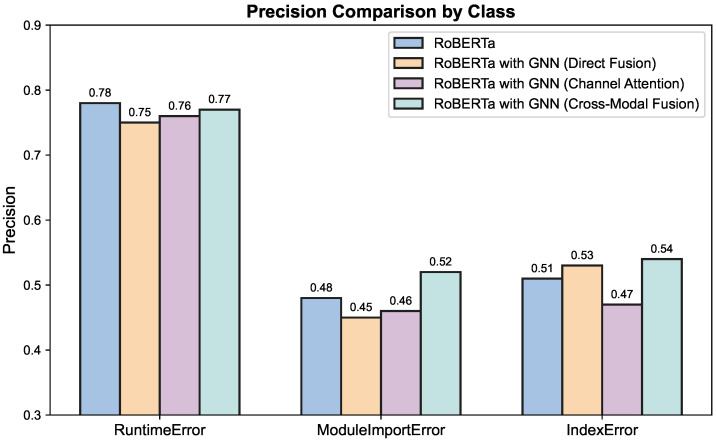
Comparison of the precision of the four models. Precision comparison across three error categories—RuntimeError (left), ModuleImportError (middle), and IndexError (right)—for four model configurations: RoBERTa, RoBERTa with GNN, RoBERTa with GNN (Channel Attention), and RoBERTa with GNN (Cross-Modal Fusion). Each group of bars represents the precision achieved by the respective models on each error type. GNN: Graph Neural Network.

As shown in Figure 2 and Table 1, the RoBERTa baseline model performs well overall, with a testing accuracy of 69.35% and a Macro F1 score of 57.34%. After introducing GNN, although the model’s representational capacity was structurally enhanced, the overall accuracy decreased slightly, and the Macro F1 score dropped significantly by about 5.05%. This performance degradation may be due to the introduction of noise when structural features are not fully optimized in conjunction with semantic information.

As compared to that, the cross-modal fusion model introduced in this paper combines semantic and structural features in an attention mechanism that surpasses all the models with the best testing accuracy of 71.06% and maintaining a Macro F1 score of 57.27%, proving that the cross-modal fusion has improvement in the model’s resilience to various kinds of errors.

**Figure 3:**
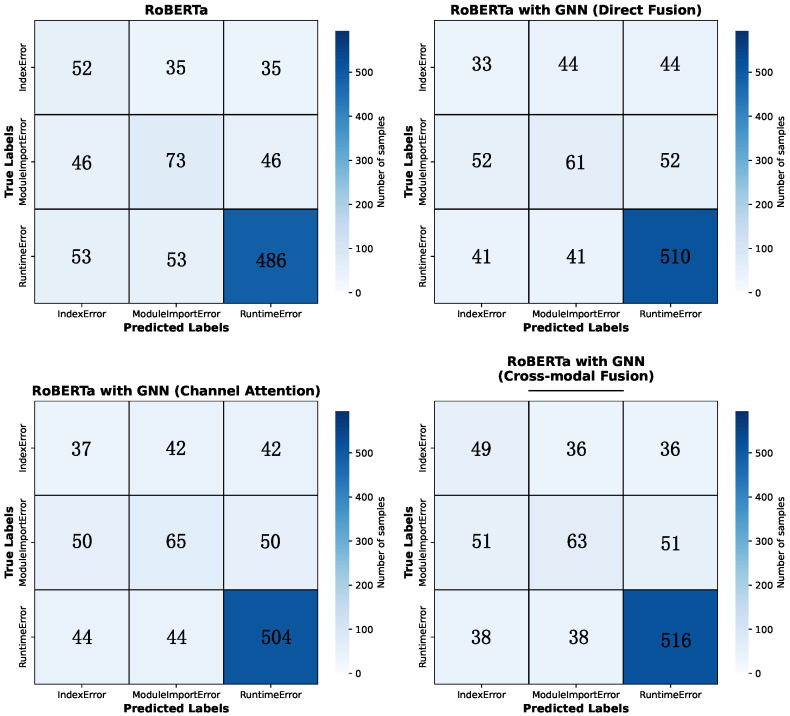
Confusion Matrix of the four models. Confusion Matrix of the four models. Confusion matrix of four models on the error type classification task, including RoBERTa (upper left), RoBERTa with GNN with direct fusion (upper right), RoBERTa with GNN with channel attention applied (bottom left), and RoBERTa with GNN with cross-modal fusion (bottom right). The horizontal axis represents the predicted categories, and the vertical axis represents the true categories. The values along the diagonal indicate the number of correctly classified samples. GNN: Graph Neural Network.

From the confusion matrix in Figure 3 of the fusion model, it can be seen that the RoBERTa baseline model performs well on Runtime Error, but there is significant confusion between Index Error and Module Import Error, indicating that the model has limited semantic ability to distinguish between these two types of errors. The RoBERTa with GNN model severely misjudges on IndexError and often predicts it as a Runtime Error, indicating that relying solely on structural features may weaken its ability to capture fine-grained semantic clues. In contrast, the model with the cross-modal fusion technique performs more evenly on various types of errors, resulting in a decrease in the overall misclassification rate, while maintaining high accuracy for Runtime Errors, it also enhances the ability to recognize Index Errors, verifying the effectiveness of semantic and structural information fusion.

Overall, confusion matrix analysis indicates that semantic information plays a key role in distinguishing errors, while structural features can play a beneficial complementary role under the premise of reasonable fusion. The fusion model demonstrates stronger category discrimination ability and lower misclassification rate, further confirming the advantages of cross-modal representation learning.

**Figure 4:**
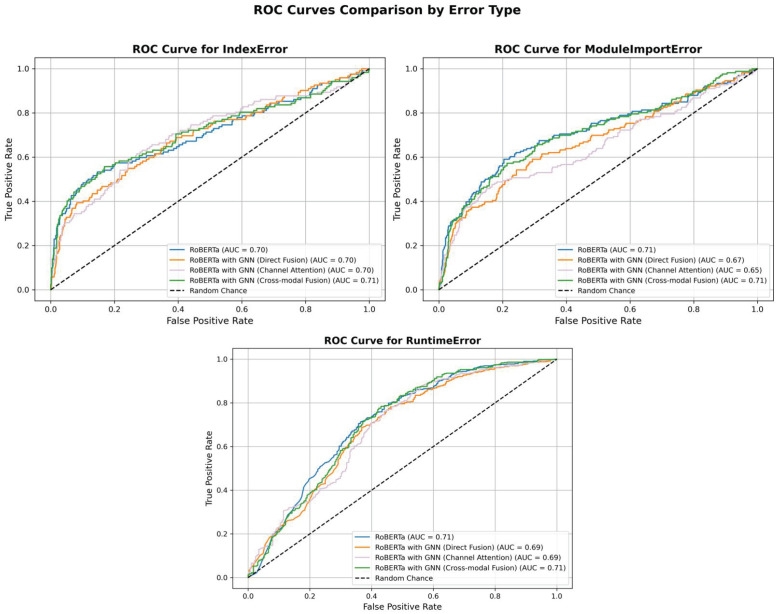
ROC Curves of the four models. ROC Curves of the four models. ROC curves for three error types (IndexError, ModuleImportError, RuntimeError) comparing RoBERTa (light blue line), RoBERTa with GNN with direct fusion (light orange line), RoBERTa with GNN with channel attention applied (light purple line), and RoBERTa with GNN with cross-modal fusion (light green line). The cross-modal model shows the best or equal AUC across all categories. ROC: Receiver Operating Characteristic; AUC: Area Under the Curve; GNN: Graph Neural Network.

To further evaluate the discriminative ability of the model, we employed the one-vs-rest strategy to compute and compare the Receiver Operating Characteristic (ROC) curves and their corresponding Area Under the Curve (AUC) values for each error category. As shown in Figure 4, among all categories, the ROC curves of the model with cross-modal fusion are generally closer to the upper left corner of the plot, indicating a better trade-off between true positive rate and false positive rate. For the classification of Runtime Errors, the AUC values of all four models are high, which is consistent with the previously reported F1 scores, suggesting that the models exhibit high confidence and consistency in identifying such errors. In contrast, the AUC scores for IndexError and ModuleImportError are comparatively lower. Notably, the model with GNN only achieve much lower AUC on IndexError, which is consistent with its lower F1 scores. The improved AUC in these categories by the cross-modal fusion model reveals that semantic and structural representation fusion can enhance the discriminative confidence of the model in handling more challenging classification tasks.

These findings suggest that semantic features play a dominant role in the identification of easily recognizable errors such as Runtime Error, while structural features serve as a complementary role in the classification of more complex categories.

## 5. Discussion

In this study, the proposed cross-modal fusion model demonstrates clear advantages in Python error classification tasks. Quantitatively, the model achieves 71.06% accuracy, outperforming the best baseline model that only use LLM. Transformer-based approaches excel in semantic feature extraction but struggle with structural dependencies, while GNN-based approaches model structural relations effectively but cannot fully capture natural language patterns embedded in error messages. The proposed cross-modal fusion mechanism leverages the complementary strengths of both modalities, reducing systematic misclassification. The confusion matrix analysis verifies this improvement, the model with cross-modal fusion showing a reduction of misclassification across all of the classes compared with model with other fusion techniques.

This study has several limitations that point toward promising directions for future research. Firstly, the training and evaluation of the proposed model are conducted exclusively on the PyTraceBugs dataset. Since the model’s performance is closely tied to the characteristics of these dataset, its adaptability to other error classification scenarios or different types of program analysis tasks remains uncertain. Future work should explore applying or fine-tuning the model on additional datasets and related classification problems to better assess its robustness and generalization ability across diverse code environments. Secondly, the model relies heavily on high-quality labeled data for training.This dependence limits the model’s scalability and may hinder its performance in low resource tasks. Future research can explore semi-supervised technique to reduce the reliance on labeled data and improve generalization under limited supervision. Finally, future work may explore the use of different graph neural network architectures on these dataset to better understand their structural modeling capabilities and suitability for code error classification. Further investigation into fusion techniques between GNNs and LLMs may enable the model to more effectively leverage complementary semantic and structural information.

## 6. Conclusions

In this article, we introduce a cross-modal fusion model for classifying errors in code. The model fuses the semantic features extracted by the RoBERTa model and the code structure information modeled by GNNs. This model is aimed to simultaneously capture natural language patterns and their structural dependencies in code, thereby enhancing the ability to identify error types.

We conducted extensive experiments on Python error datasets to validate the effectiveness of the proposed model. Experimental results show that the proposed model achieves the highest performance among all evaluated baselines, reaching 71.06% test accuracy and a 57.27 macro-F1 score. It outperforms the best single-modality model by 1.75% and surpasses all other fusion-based approaches. This model demonstrates higher discriminative ability especially on semantically similar and difficult distinguish error types such as IndexError and ModuleImportError. Through the analysis of the confusion matrix and ROC-AUC curve of the models, it can be concluded that cross-modal fusion not only improved the model’s ability to distinguish various errors in code, but also reduced systematic misclassification, indicating that the fusion of semantic and structural information has a positive impact on the model’s robustness and generalization ability.

This study suggests that a reasonable and refined fusion mechanism is crucial for achieving synergistic gains between LLM and GNN. Only with specific and appropriate fusion methods can the model have advantages in the field of code error detection. The design of the fusion strategy should fully consider the differences in structure, semantics, and information transmission mechanisms between the two types of models, promoting complementary and adaptive features.

## Figures and Tables

**Table 1:** Evaluation of performance of the four models.

Model	Test Accuracy	Macro-Averaged F1 Score
RoBERTa	69.35	57.34
RoBERTa with Graph Neural Network (Direct Fusion)	68.79	52.29
RoBERTa with Graph Neural Network (Channel Attention)	69.01	53.33
RoBERTa with Graph Neural Network (Cross-Modal Fusion)	71.06	57.27

## Data Availability

The data presented in this study are available on request from the corresponding author due to privacy and confidentiality restrictions of the laboratory.
